# New Poly(lactic acid) Active Packaging Composite Films Incorporated with Fungal Melanin

**DOI:** 10.3390/polym10040386

**Published:** 2018-04-01

**Authors:** Łukasz Łopusiewicz, Filip Jędra, Małgorzata Mizielińska

**Affiliations:** Center of Bioimmobilisation and Innovative Packaging Materials, Faculty of Food Sciences and Fisheries, West Pomeranian University of Technology Szczecin, Janickiego 35, 71-270 Szczecin, Poland; filip.jedra@zut.edu.pl (F.J.); malgorzata.mizielinska@zut.edu.pl (M.M.)

**Keywords:** poly(lactic acid), biopolymer, melanin, packaging, polymer blends, antioxidant, barrier properties, mechanical properties

## Abstract

In this work, fungal melanin was used for the first time to prepare poly(lactic acid)-based composites. The films of various melanin concentrations (0.025%, 0.05% and 0.2% *w*/*w*) were prepared using an extrusion method. The mechanical, antioxidant, antimicrobial, water vapor and UV-Vis barrier properties, as well as available polyphenolics on the surface, were studied. FT-IR and Raman spectroscopy studies were carried out to analyze the chemical composition of the resulting films. The hydrophobicity, color response, thermal, optical properties, and opacity values were also determined. The results of this study show that the addition of fungal melanin to poly(lactic acid) (PLA) as a modifier influenced mechanical and water vapor barrier properties depending on melanin concentration. In low concentration, melanin enhanced the mechanical and barrier properties of the modified films, but in larger amounts, the properties were decreased. The UV-Vis barrier properties of PLA/melanin composites were marginally improved. Differential Scanning Calorimetry (DSC) analysis indicated that crystallinity of PLA increased by the addition of melanin, but this did not affect the thermal stability of the films. Modified PLA/melanin films showed good antioxidant activity and were active against *Enterococcus faecalis*, *Pseudomonas aeruginosa* and *Pseudomonas putida*. The addition of melanin caused changes in color values, decreasing lightness and increasing the redness and yellowness of films. Based on the results of this study, fungal melanin has good potential to be exploited as a value-added modifier that can improve the overall properties of PLA.

## 1. Introduction

Natural polymers, biopolymers, and synthetic polymers based on annually renewable resources are the basis of a 21st-century portfolio of sustainable, eco-efficient plastics. These biosourced materials are hoped to gradually replace the currently existing family of petroleum-based polymers as they become less competitive regarding cost performance [1,2]. Biodegradable polymers from renewable resources have attracted a large amount of attention in research. They are defined as polymers that undergo microbially-induced chain scission leading to mineralization [3,4]. Polylactide or poly(lactic acid) (PLA) is the most promising bio-based polymer in the emerging bioplastics market, with the high availability and a more attractive cost structure. PLA has a panoply of advantages: it is a thermoplastic material with a rigidity and clarity similar to polystyrene (PS) or poly(ethylene terephthalate) (PET), it is bio-based, resorbable and biodegradable under industrial composting conditions, but also processable in most standard equipment (injection molding, blow molding, thermoforming, extrusion and casting [1,3,5,6,7,8]. PLA has a wide spectrum of applications: packaging, medical, agricultural and engineering materials, as well as textile preparation [1].

The end uses of PLA within the packaging industry include rigid packaging, flexible film packaging, cold drinks cups, cutlery, apparel and stable fibers, bottles, injection molded products, as well as extrusion coatings [1,8]. PLA also has a wide spectrum of medical applications, such as surgical implant materials, dental materials, drug delivery systems, guided tissue and bone regeneration platforms, porous scaffolds for tissues growth and fracture fixation devices [4,7,9].

As a packaging material PLA is attractive because it exhibits a tensile strength comparable to that of petroleum-derived thermoplastics, is biodegradable and can be sealed at low temperatures [1]. The properties of PLA, such as thermal stability and impact resistance, medium gas barrier properties and low solvent resistance of pure PLA are inferior to those of conventional polymers used for thermoplastic applications. Therefore, PLA is not ideally suited to compete against conventional polymers and limits its use for food packaging applications [10]. In order to improve the properties of PLA and increase its potential application, PLA modification, copolymerization with other monomers, and PLA composites are still being developed to improve its properties, regarding stiffness, permeability, crystallinity, and thermal stability [1].

A wide spectrum of additives (nano or microsized) have been used for the preparation of PLA composites including: clays and organoclays [11,12], starch [5], carbon nanotubes [13], metal nanoparticles [14,15,16], graphene [7,13,17], as well as cellulose and glass fibers [18], aromatic compounds and essential oils [19,20], chitosan [21,22], epoxidized vegetable oils [10], plant fibers (wood [23], bamboo [24], banana [25], cotton and flax [26], hemp [27] and artichoke [28]), collagen [9] and tannins [29]. Those additives may not only influence the mechanical, optical properties and thermal stability of the blends but also enhance the microbial stability of the packaged foods by using active packaging developed by the incorporation of antimicrobial compounds into the polymer matrix. Contact between the active materials and food, which has the ability to change food composition or the atmosphere around it, represents an active packaging system that inhibits the growth of microorganisms present on the surface of food products [30,31]. PLA could also be modified by active coatings that contain antimicrobials [32].

Melanins have been isolated and characterized from a variety of phylogenic sources, such as animal [33], plant [34], bacteria [35] and fungi [36,37]. Melanins are commonly represented as black and brown pigments, high molecular weight heterogeneous polymers derived from the oxidation of monophenols and the subsequent polymerization of intermediate o-diphenols and their resulting quinones [38]. Melanins are types of pigments, possessing broad biological properties including antioxidant, radioprotective, thermoregulative, chemoprotective, antitumor, antiviral, antimicrobial, immunostimulating and anti-inflammatory [33,34,35,36,37,38]. Potentially, these melanin attributes could also be imparted to plastics, and in the case of bioplastics, potentially enhancing performance as well as sustainability credentials, explore its other nonconventional applications such as crosslinking during polymerization, antioxidant and antimicrobial activity, radioprotective ability and improving the biological properties of the polymer. The use of melanins remains relatively unexplored with few examples of these compounds blended with plastics [39,40,41], and no studies on PLA/melanin composites are available in the scientific literature.

The aim of this study was to investigate the influence of fungal melanin on the properties of modified poly(lactic acid) films. To evaluate the potentiality of the developed films for different industrial and biomedical applications, the mechanical, barrier, antioxidant and antimicrobial properties were all evaluated. Spectroscopic studies were performed to elucidate melanin addition in the chemical composition of modified blends. The goal of the study was also to evaluate the influence of fungal melanin on the color, opacity and optical properties of the films.

## 2. Materials and Methods

### 2.1. Poly(lactic acid)

Poly(lactic acid) (PLA), 4043D, was purchased in pellet form from Resinex (a Polish supplier of Natureworks resins)—dedicated to extruding thin films through a casting method. 

### 2.2. The Isolation of Melanin from A. bisporus Waste

Agricultural waste from the production of *A. bisporus* (ABW—Agaricus Bisporus Waste) in the form of fruiting bodies stipes was obtained from a local producer in Wolsztyn (Wielkopolskie voivodeship, Poland). 500 g of ABW was first homogenized (Heidolph Brinkmann Homogenizer Silent Crusher, Schwabach, Germany) in 500 mL of distilled water and incubated (24 h, 37 °C) to allow enzyme tyrosinase action (hydroxylation of monophenols to *o*-diphenols). After incubation, the homogenate mixture was adjusted to pH = 10 by 1 M NaOH, and incubated (24 h, 65 °C) to allow a spontaneous oxidative polymerization of the resulting *o*-diphenols and quinones to form melanin. Afterward, the mixture was filtered, centrifuged (6000 rpm, 10 min), and an alkaline ABW raw melanin mixture was used to purify the melanin. An alkaline ABW raw melanin mixture was first adjusted to pH 2.0 with 1 M HCl to precipitate melanin, followed by centrifugation at 6000 rpm for 10 min and a resulting pellet was collected. The pellet was then hydrolyzed in 6 M HCl (90 °C, 2 h), centrifuged (6000 rpm, 10 min) and washed in distilled water five times to remove acid. The pellet was washed with chloroform, ethyl acetate and ethanol three times to wash away lipids and other residues. Finally, the purified melanin was dried, ground to a fine powder in a mortar and stored at −20 °C until testing.

### 2.3. The Preparation of PLA/Melanin Films

The blends of PLA and melanin were obtained by means of a twin-screw extruder combined with an air-cooling system and a side-cut pelletizer which feature a co-rotating twin-screw extruder (screws of 20 mm diameter, a length/diameter ratio (L/D): 40/1, LabTech Engineering, Samut Prakan, Thailand), cooling conveyor (equipped with 8 fans, LabTech Engineering, Samut Prakan, Thailand), side-cut pelletizer (with 8 rotary knives producing pellets with dimensions of 5 × Ø3 mm, LabTech Engineering, Samut Prakan, Thailand). An average of 75–80 micron thick films were obtained by means of a laboratory cast film extrusion line (LabTech Engineering, Samut Prakan, Thailand), with following equipment parameters: screw diameter—20 mm; length/diameter (L/D) screw ratio of: 30/1; output of extruder (each): approx. 5 kg/h of LDPE; screw type: transporting-mixing/ transporting; screw speed (rpm) during the experiment: 60. “0”—pure PLA film (devoid of melanin, served as a control sample) and three PLA/melanin composites with various concentrations of melanin were prepared: “1”—0.025%; “2”—0.05% and “3”—0.2%.

### 2.4. The Mechanical Properties of PLA/Melanin Films

Mechanical measurements were tested by the use of Zwick/Roell 2,5 Z equipment (Zwick/Roell, Ulm, Germany) and they included tensile strength (the gap between tensile clamps was 25 mm and tensile speed was 100 mm/min), and burst strength (transducer diameter 0.75 mm, speed 50 mm/min). The dynamic mechanical analyses of the pure PLA and modified PLA/melanin films were performed by the use of DMA analyzer (Q800, TA Instruments, New Castle, DE, USA). Films were prepared as a rectangular (30 mm × 5 mm × 0.08 mm) samples. Samples were located between clamps and analyzed with following parameters: temperature range 30–80 °C; constant heating rate 3 °C/min; amplitude 15 μm; force track 125%; oscillation frequency 1 Hz. The storage modulus (*E*’), loss modulus (*E*’’), loss factor (tan δ), and glass transition temperature (*T*_g_) of each specimen were obtained as a function of temperature.

### 2.5. The Water Vapor Transmission Rate of the Films

The Water Vapor Transmission Rate (WVTR) was measured by means of a gravimetric method that is based on the sorption of humidity by calcium chloride and a comparison of sample weight gain. Initially, the amount of dry CaCl_2_ inside the container was 9 g. The area of film was 8.86 cm^2^. Measurement was carried out for a period of 4 days, each day the containers were weighed to determine the amount of absorbed water vapor through the films. The result was expressed as average values from each day of measurement and each container. Analyses were carried out at ten independent containers for each type of films, calculated as a standard unit g/(m^2^ × day) and presented as a mean ± standard deviation.

### 2.6. The Contact Angle (CA)

The surface properties of modified and pure PLA films were measured through a contact angle analyzer. The following measurement was carried out by means of a laboratory goniometer (Haas μL), drop of distilled water was placed on the surface of the film using a microsyringe. Analyses were carried out at three independent times and presented as mean ± standard deviation.

### 2.7. Spectral Analysis

#### 2.7.1. UV-Vis Spectroscopy

The UV-Vis spectra of the films samples were measured by the use of a UV-Vis Thermo Scientific Evolution 220 spectrophotometer at 200–800 nm.

#### 2.7.2. FTIR Spectroscopy

Fourier transform infrared (FT-IR) spectra of the unmodified and modified film samples were measured using a FT-IR spectroscopy (Perkin Elmer Spectrophotometer, Spectrum 100, Waltham, MA, USA), operated at a resolution of 4 cm^−1^, over 64 scans. Film samples were cut into square shapes (2 cm × 2 cm) and placed directly at the ray-exposing stage. The spectra were recorded at a wavelength of 650–4000 cm^−1^. The spectra were normalized, baseline corrected and analyzed using SPECTRUM software.

#### 2.7.3. Raman Spectroscopy

Pure and modified films were analyzed using a Raman station (RamanStation 400F, Perkin Elemer, Waltham, MA, USA) with point-and-shot capability using an excitation laser source at 785 nm, 100 micron spot size. Film samples were cut into square shapes (2 cm × 2 cm) and placed directly at the ray-exposing stage. The spectra were recorded at a wavelength of 250–3300cm^−1^. The spectra were normalized, baseline corrected and analyzed using SPECTRUM software (v10, PerkinElmer, Waltham, MA, USA).

### 2.8. Color Response Analysis

The color changes of the films were measured by using a colorimeter (CR-5, Konica Minolta, Tokyo, Japan). The results were expressed as L* (lightness), a* (red to green), and b* (yellow to blue) parameters to evaluate color changes in the modified PLA/melanin films. All of the measurements were determined at three random points on both sides of each film, and the experiments were performed five times and presented as a mean ± standard deviation.

To determine other color properties of the films, ∆*E* (color difference), *YI* (yellowness index) and *WI* (whiteness index) values were calculated using following equations (where pure PLA film served as a standard):

∆*E* = [(*L*_standard_ − *L*_sample_)^2^ + (*a*_standard_ − *a*_sample_)^2^ + (*b*_standard_ − *b*_sample_)]^0.5^ (1)

*YI* = 142.86b*L^−1^ (2)

*WI* = 100 − [(100 − *L*)^2^ + *a*^2^ + *b*^2^]^0.5^ (3)

### 2.9. Opacity Measurements

The opacity of modified PLA/melanin films and pure PLA was carried out in Opacimeter EE Model 12 (Diffusion Systems Ltd., London, UK). The opacimeter was initially calibrated using standard white plate (value 100 ± 1, Diffusion Systems Ltd.) and measurements were performed on each film six times, and presented as mean ± standard deviation.

### 2.10. The Antioxidant Activity of PLA/Melanin Blends

#### 2.10.1. Determination of Available Phenolic Groups on the Modified Films Surface

The method for the determination of available phenolic groups (APG) on the modified PLA/melanin films was carried out according to Bishai et al. [42]. 100 mg of PLA/melanin films or pure PLA was taken in a volumetric flask. Sequentially, 1 mL of 10% Folin-Ciocalteu reagent and 4 mL of 2% sodium carbonate solution were added to the flask. Finally, the volume was made up to 25 mL with distilled water and mixed well. The reaction mixture was kept at room temperature for 48 h and the resultant absorbance was determined at 760 nm. A control absorbance was also measured where the aforesaid reaction mixture, devoid of any film, was kept under the same reaction conditions. To determine the available phenolic groups on the modified films surface, a calibration curve was prepared using gallic acid standard solutions and the results were expressed as μmoles of gallic acid equivalents (GAE) per gram of dry film. All experiments were performed in triplicate and presented as mean ± standard deviation.

#### 2.10.2. A Determination of the Free Radical Scavenging Activity of Modified Films

The free radical scavenging property determination of melanin incorporated PLA films was carried out using ABTS and DPPH reagents according to Bishai et al. [42]. Radical 2,2′-azino-bis(3-ethylbenzothiazoline)-6-sulphonic acid (ABTS^+^) was produced by mixing 7 mM ABTS with 2.45 mM potassium persulfate (5 mL of ABTS + 5 mL of potassium persulphate 4.9 mM). The mixture was then incubated for 16 h in the dark, at room temperature and subsequently diluted with water to an absorbance of maximum 1.00 at 734 nm. To determine the antioxidant capacity of PLA/melanin films, 1 g of the film was put into 25 mL of ABTS^+^ solution and incubated up to 24 h at room temperature. Control sets without the film were also kept under identical conditions. After incubation period, the film samples were removed from the ABTS^+^ solution. Absorbance for both sets was taken and antioxidant activity (*AA*%) was calculated using the equation:

*AA*% = [(*A*_control_ − *A*_sample_)/*A*_control_] × 100 (4)

1 g of modified PLA/melanin films was taken in 25 mL of 0.004% (*w*/*v*) methanolic DPPH solution. *A* set of controls was also placed where 25 mL of the same solution was taken, excluding the addition of the film. Both samples were incubated up to 24 h, at room temperature. After incubation period, the film samples were removed from the DPPH solution. Sample and control set absorbance were measured at 517 nm and the free radical activity of the polymeric films was calculated according to the same equation as the ABTS method.

### 2.11. The Antimicrobial Activity of Films

The test microorganisms used in this study were obtained from the American Type Culture Collection (ATCC). The strains used in this study were *Escherichia coli* ATCC8739, *Enterococcus faecalis* ATCC29212, *Pseudomonas aeruginosa* ATCC2783, *Pseudomonas putida* ATCC31753 and *Staphylococcus aureus* ATCC12600. To verify the antimicrobial properties of films Mueller-Hinton medium (Merck, Darmstadt, Germany) was used. The medium was prepared according to the Merck protocol (medium was weighted according to the manufacturer’s instructions, suspended in 1000 mL of distilled water, and autoclaved at 121 °C for 15 min). The film samples were cut into square shapes (5 cm × 5 cm). The antimicrobial properties of non-modified and modified films were carried out according to ISO 22196:2007(E) standard [43].

### 2.12. Microscopic Examination of Films

To determine the melanin particles distribution in resulted films the samples were examined under the light microscope Zeiss SteREO Discovery.V20 (Carl Zeiss Microscopy GmbH, Jena, Germany)equipped with PlanApo S lens (2.3×, FWD 10 mm) and AxioCam MRc 5 camera, with magnitude 17.2× and 3.7 μm resolution.

### 2.13. Differential Scanning Calorimetry (DSC)

DSC measurements were performed using a DSC calorimeter (204 F1 Phoenix, NETZSCH, Selb, Germany) in a temperature range from −25 °C to 210 °C at φ = 5 °C/min, performing two heating and one cooling scans. Melting and cold crystallization temperatures and enthalpies (*T*_m_, *T*_c_, Δ*H*_m_, Δ*H*_c_) were determined from the second heating scan and glass transition temperatures (*T*_g_) were also measured. The crystallinity degree (*χ*) was calculated according to the following Equation:

*χ*(%) = (Δ*H*_m_/*W*Δ*H*_m0_) × 100 (5)

where Δ*H*_m_ is the enthalpy for melting, Δ*H*_m0_ is enthalpy of melting for a 100% crystalline PLA sample (taken as 93 J/g) and *W* is weight fraction of PLA in the sample [16].

All the experiments were carried out under nitrogen flow. The results were visualized and analyzed using NETZSCH Proteus Software (v6.0, NETZSCH-Gerätebau GmbH, Selb, Germany).

### 2.14. Statistical Analyses

All determinations were carried out in triplicate as a minimum. Statistical significance was determined using an analysis of variance (ANOVA) followed by Duncans’s test. The values were considered as significantly different when *p* < 0.05. All analyses were performed with Statistica version 10 (StatSoft Polska, Kraków, Poland).

## 3. Results

### 3.1. Material Processing

The process of extrusion was even and stable and no “disturbing” effects regarding the addition of melanin in a form of powder were observed. All parameters, such as pressure and motor current remained at typical values for PLA. The process of compounding the composite was preceded by drying according to the technical data sheet.

### 3.2. Mechanical Properties

Table 1 shows that the ratios 0.025% and 0.05% were able to slightly improve the tensile strength of the PLA-based films in a transversal direction (*p* < 0.05). It was observed that the highest concentration of melanin (0.2%) led to a decrease in tensile strength in both directions, as well as a burst strength in the PLA-based films (*p* < 0.05). The results obtained for the other two concentrations were comparable to the results of the reference film. With regards to the burst strength of the samples, the lowest (sample “1”) addition of melanin to the polymer matrix increased the maximum force value slightly—from 23.50 ± 2.41 MPa to 27.45 ± 1.45 MPa. Sample “3” exhibited the lowest value, whereas sample “2” had an almost identical value to the reference sample “0” (*p* > 0.05). All the other differences between the results were statistically significant as proved by the Duncan test that *p* < 0.05.

Due to the powder-like, non-thermoplastic characteristics of melanin it had to be taken into consideration that the additive could have deteriorated the sealing behavior of the PLA-based composite films. However, only the greatest addition (0.2%) of melanin resulted in a significant decrease in the sealing strength—from 10.18 ± 1.68 MPa to 7.12 ± 0.77 MPa (*p* < 0.05). The differences between the reference value and those obtained for samples “1” and “2” were not statistically significant (*p* > 0.05).

**Figure 1:**
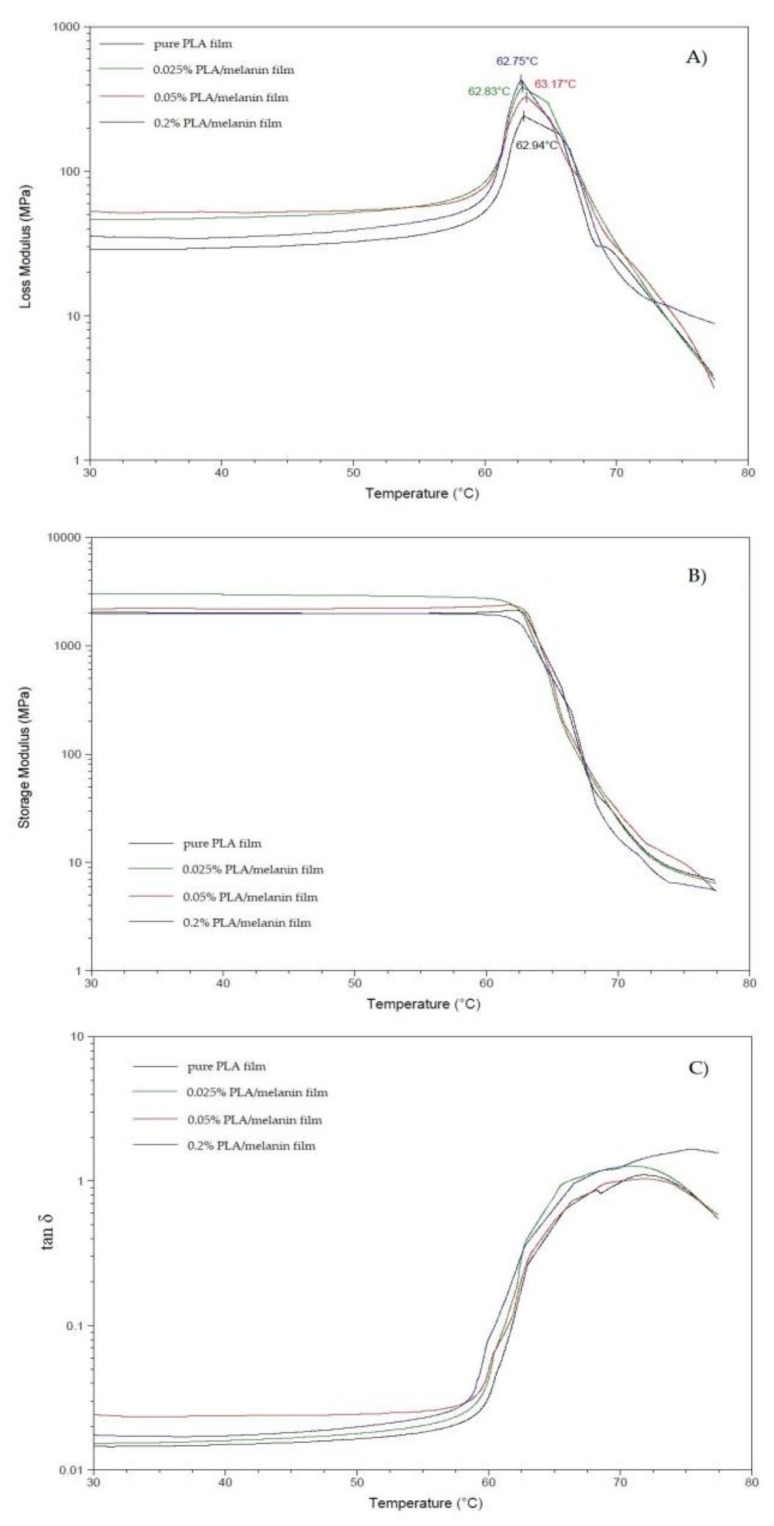
Loss modulus (**A**); storage modulus (**B**) and tan δ (**C**) of pure poly(lactic acid) (PLA) and PLA/melanin modified films.

As concerns the dynamic mechanical analysis (DMA), the temperature curves of the storage modulus (*E*’), loss modulus (*E*’’) and of the loss factor (tan δ) are shown in Figure 1. The storage modulus of the sample “1” and “2” at medium temperatures is higher than that of the pure PLA film, probably because of the reinforcement effect of melanin particles. These data are in agreement with the tensile strength results, as discussed above: the addition of melanin at concentrations 0.025% and 0.05% reinforce the PLA matrix in contrast to concentration 0.2%. As shown in Figure 1B, the storage modulus decreases by increasing the temperature for all of the samples, with a significant drop in the range between 60 °C and 70 °C, corresponding to the glass transition region. Figure 1C shows also the loss modulus of pure PLA and PLA/melanin modified films. The glass transition temperature (*T*_g_) evaluated as the temperature at which the damping attains its maximum value is shown in Figure 1. In particular, the addition of melanin influenced slightly the *T*_g_ temperature and those results were not statistically significant (*p* > 0.05).

### 3.3. Surface Properties—Contact Angle

Three repetition tests were performed for each sample of the PLA-based films. The average values of the contact angle obtained for distilled water were as follows: 66.96°; 67.67°; 67.00°; 67.25° for samples “0”, “1”, “2”, and “3”, respectively. The Duncan’s test was applied to demonstrate that these differences of averaged values were statistically insignificant (*p* > 0.05).

### 3.4. Barrier Properties—WVTR

Polylactide itself is not a high barrier material, both in terms of transmission of oxygen and water vapor. The water vapor transmission rate of all four samples was measured by means of a gravimetric method, which is based on the sorption of humidity by calcium chloride and a weight gain comparison of the samples. As reported in the Table 1 sample “1” exhibited the lowest values of WVTR which was 21.30 ± 2.56 g/(m^2^ × day), whereas the reference value was 24.60 ± 0.33 g/(m^2^ × day). However, after considering standard deviations and statistical analysis, these differences are not statistically significant. Sample “2” had a comparable value with the reference value and sample “3” exhibited the highest value of 28.20 ± 1.31 g/(m^2^ × day). As before, Duncan test was carried out in order to verify the differences between WVTR values. This was calculated by Statistica software that following differences: “0” vs. “1”, “0” vs. “2” and “0” vs. “3” were statistically significant (*p* < 0.05). All of the other pairs of average values were not statistically significant (*p* > 0.05).

### 3.5. The Spectral Analysis of Modified Films

#### 3.5.1. UV-Vis Spectra

**Figure 2:**
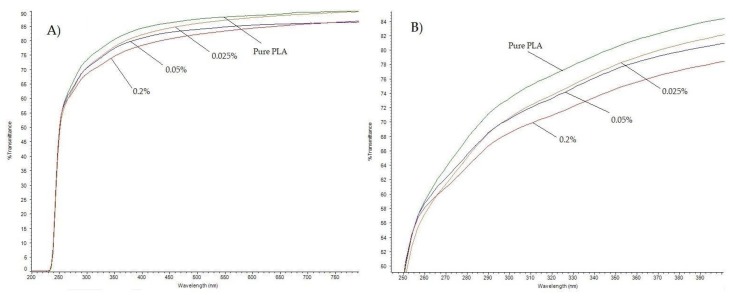
The UV-Vis spectra of pure PLA and PLA/melanin modified films at 200–800 nm (**A**) and 250–400 nm (**B**).

UV-Vis spectra of pure PLA and PLA/melanin films are shown in Figure 2. The addition of melanin caused moderate improvement of the light barrier properties. Sample “3” showed approximately 7–8% lower transmittance values at UV-A and UV-B regions. From 250 nm all samples showed the same transmittance pattern.

#### 3.5.2. The FT-IR Spectra

**Figure 3:**
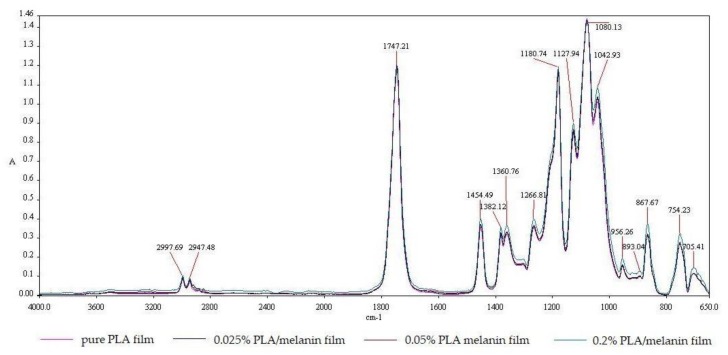
The FT-IR spectra of pure PLA and PLA/melanin modified films.

The FT-IR spectra of pure PLA and PLA/melanin films are shown in Figure 3. The strong IR bands at 2997.69 cm^−1^; 2947.48 cm^−1^ are assigned to the -CH stretching region (–CH_3(asymm)_, –CH_3(symm)_, and –CH modes). The C=O stretching region appeared in IR spectra at about 1747.21 cm^−1^ as a broad asymmetric band mainly due to A and E_1_ active modes. –CH_3_ was responsible for the appearance of the band at 1454.49 cm^−1^. –CH deformation and asymmetric bands appeared at 1382.12 cm^−1^ and 1360.76 cm^−1^. Moreover, the –CH bending modes resulted in bands at 1315 cm^−1^ and 1300 cm^−1^. The C–O stretching modes of the ester group appeared at 1266.81 cm^−1^ and the C–O–C asymmetric mode appeared at 1080.13 cm^−1^. At 956.26 cm^−1^, bands characteristic of helical backbone vibrations with CH_3_ rocking modes were visible. At 867.67 cm^−1^ and 754.23 cm^−1^ two bands appeared that could be attributed to the amorphous and crystalline phases of PLA, respectively [1]. These peaks can be also assigned to –C–C– stretching and C=O stretching, respectively [16]. Only the spectrum of PLA/melanin film with the highest melanin content (sample “3”) offered noticeable differences, mainly at wavelengths: 1454.49 cm^−1^; 1382.12 cm^−1^; 1360.76 cm^−1^; 1127.94 cm^−1^; 1042.93 cm^−1^; 956.26 cm^−1^; 867.67 cm^−1^; 754.23 cm^−1^ and 705.41 cm^−1^. Those differences were the result of melanin addition into PLA.

#### 3.5.3. The Raman Spectra

**Figure 4:**
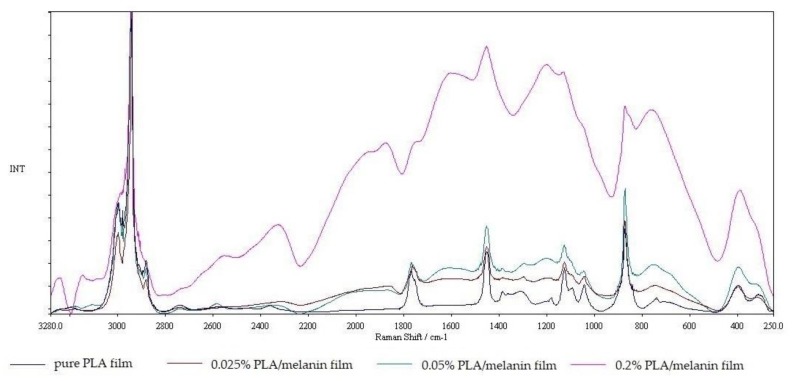
The Raman spectra of pure PLA and PLA/melanin modified films.

Raman spectroscopy of pure PLA and modified PLA melanin films can be seen in Figure 4. Pure PLA spectra can be characterized with stretching –C=O groups which are present at many wave number values. Weak –C=O groups are present at a frequency range from 675 cm^−1^ to 711 cm^−1^ while moderate groups can be found between 736 cm^−1^ and 760 cm^−1^. Strong –C=O groups in PLA polymer can be found at 1773 cm^−1^. –C=O groups broadened at a frequency range from 1700 to 1800 cm^−1^. Specific peak group of PLA polymer appeared at 1450 cm^−1^ which is attributed to asymmetric –CH groups. –CH_3_ symmetric group used to appear at a frequency range between 1384 and 1388 cm^−1^ shifted to 1390 cm^−1^. Then, –CH_3_ asymmetric groups are found at 1128 cm^−1^. Raman spectra at a frequency range of 1179 and 1216 cm^−1^ were moderate –C–O–C asymmetric groups.

### 3.6. The Visual Appearance and Color

**Figure 5:**
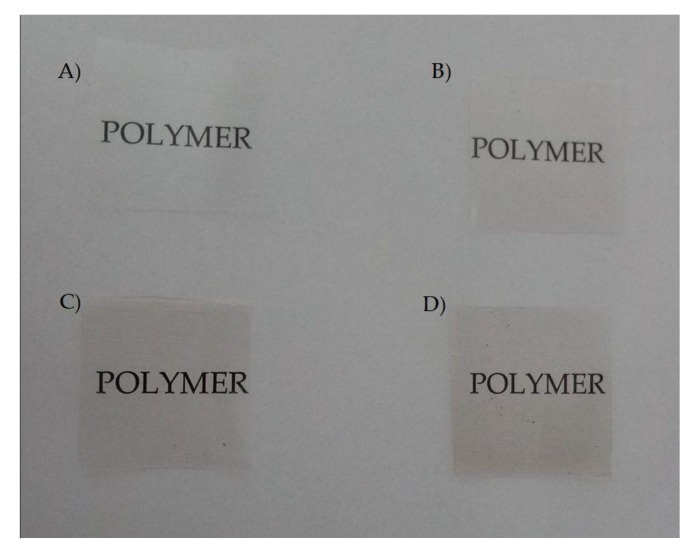
The visual appearance of the films: (**A**) pure PLA film (**B**) 0.025% PLA/melanin film (**C**) 0.05% PLA/melanin film (**D**) 0.2% PLA/melanin film.

**Figure 6:**
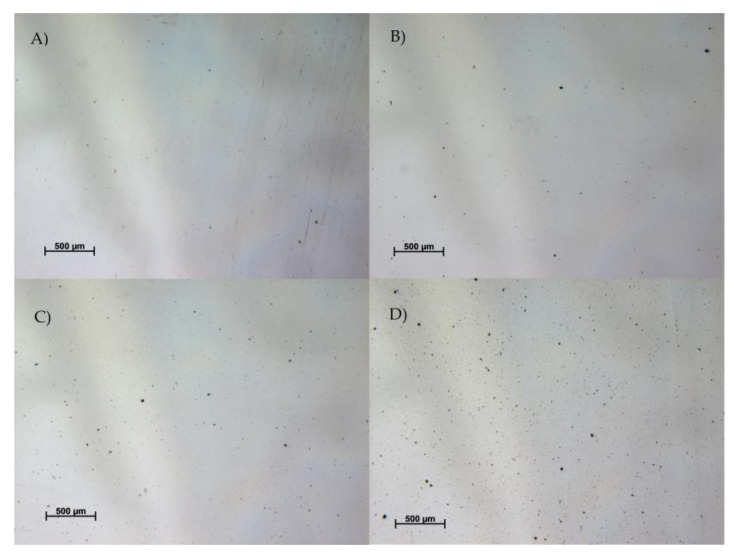
The results of microscopic examination of the films: (**A**) pure PLA film (**B**) 0.025% PLA/melanin film (**C**) 0.05% PLA/melanin film (**D**) 0.2% PLA/melanin film.

The visual appearance of pure PLA and PLA/melanin modified films is shown in Figure 5 while the results of microscopic examination are shown in Figure 6. As can be seen in Figure 6 the distribution of melanin particles in polymer matrix was homogenous. The majority of melanin particles size was less than 1 μm. The color, ∆*E*, *YI* and *WI* values are presented in Table 2. The growing addition of melanin influenced the color values in comparison to pure PLA film, causing a reduction in the lightness (L*) and an increase in the redness (a*) and yellowness (b*) values. Color differences were statistically significant (*p* < 0.05). ∆E values ranged from 0.60 (sample “1”) to 1.33 (sample “3”). The yellowness (*YI*) increased with increasing melanin amount, in contrast, the whitening index (*WI*) decreased when the melanin content was increasing.

### 3.7. Opacity

The opacity of pure PLA and PLA/melanin modified films is shown in Table 2. The opacity values of modified PLA/melanin films were lower than the pure PLA film. The opacity of PLA/melanin films decreased after the addition of melanin particles (from 6.9 ± 0.09 of sample “0” to 6.41 ± 0.12 of sample “3”). This may have been due to the color and the content of the melanin particles. Those differences were not statistically significant (*p* > 0.05).

### 3.8. Antioxidant Activity

Table 3 presents results of an assessment of the available phenolic groups on the films surface and antioxidant activity of pure PLA and PLA/melanin modified films. The total available phenolics were determined to be 0.018; 0.020 and 0.033 μmole GAE/g film for samples “1”, “2” and “3”, respectively. No polyphenolics were detected in the pure PLA film (sample “0”). The antioxidant activity of modified PLA/melanin films grew with the increasing content of melanin, reaching 21.66% and 23.20%, which was determined by DPPH and ABTS methods, respectively. No antioxidant activity of the unmodified PLA film was observed. Differences between the modified films and pure PLA were statistically significant (*p* < 0.05).

### 3.9. The Crystallization Characterization—DSC

**Figure 7:**
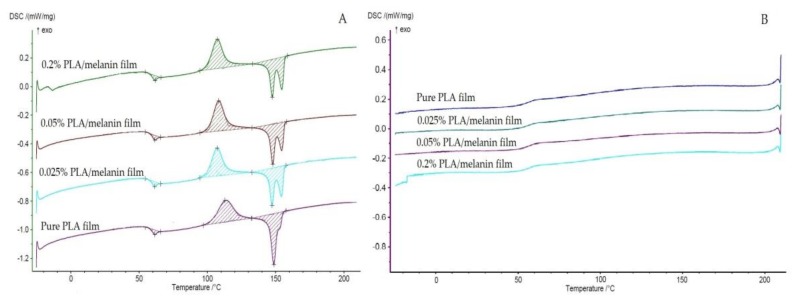
The DSC curves of pure PLA and PLA/melanin modified films (**A**) second heating scan (**B**) cooling.

In this study, DSC measurements were carried out to investigate the thermal characteristics of the films. The glass transition temperatures (*T*_g_), cold crystallization temperatures (*T*_c_), melting temperatures (*T*_m_) and crystallinity degrees (*χ*) of the tested materials at φ = 5 °C/min are summarized in Table 4. The thermograms corresponding to the second heating scan (A) and cooling (B) of pure PLA and PLA/melanin modified films are shown in Figure 7. Compared to the pure PLA film, the addition of melanin particles did not significantly affect the *T*_g_ and *T*_m_ (*p* > 0.05) of the modified PLA/melanin films. *T*_g_ of films resulted from DSC are comparable to the results of DMA analysis. The PLA/melanin films displayed double melting behavior. It can be seen that the degree of crystallinity (*χ*) of pure PLA was 0.23%, while crystallinity degrees of samples “1”, “2” and “3” were 0.92%; 2.43%; 1.61% respectively.

### 3.10. Antimicrobial Activity

**Figure 8:**
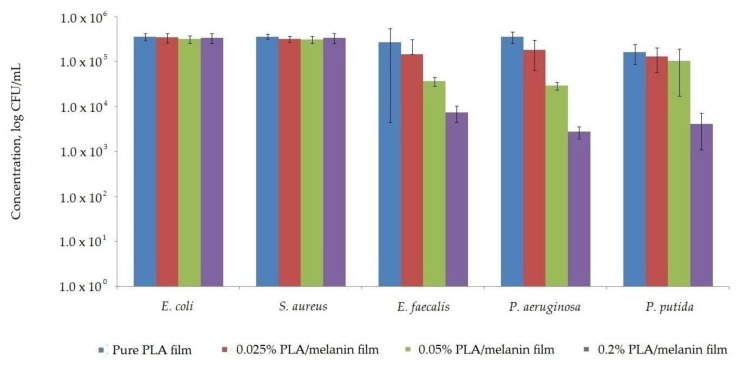
The influence of pure PLA and PLA/melanin modified films on *E. coli*, *S. aureus*, *E. faecalis*, *P. aeruginosa* and *P. putida* growth.

The susceptibility assay of *E. coli*, *E. faecalis*, *P. aeruginosa*, *P. putida* and *S. aureus* with respect to the pure PLA and modified PLA/melanin films is shown in Figure 8. The results of this research determined that neither pure PLA nor modified films were found to be active against *E. coli* and *S. aureus*. As indicated by statistical analysis, the differences between numbers of *E. coli* and *S. aureus* cells exposed to pure PLA and modified PLA/melanin films were not significant (*p* > 0.05). The results of this research demonstrated that pure PLA films had no influence on the growth of *E. faecalis*, *P. aeruginosa* and *P. putida* cells but the cells exhibited sensitivity towards modified films. A log reduction of the number of cells was noted when bacterial cells were exposed to highest melanin concentration in modified films. As can be seen in Figure 8 the growth of *E. faecalis*, *P. aeruginosa* and *P. putida* were observed after contact with samples “1” and “2”, but the number of bacterial cells was reduced than in comparison to control film, devoid of melanin. The differences between the number of viable cells were significant, as confirmed by a Duncan test (*p* < 0.05).

## 4. Discussion

The results of this study presented that the melanin isolated from ABW used as an additive for PLA at various concentrations may influence the properties of modified films depending on concentration. The increased availability of PLA has stimulated increased research and development activities, which can also be partly attributed to the escalating “green” movement that is encouraging the use of bio-polymers. Since the packaging industry plays a dominant role in the short-term use of cheap non-biodegradable petroleum-based materials, their replacement with PLA could provide a significant step to eco-friendly solutions [1,5,44]. Hence, the incorporation of melanin has opened up new avenues to discover its applicability in the packaging industry, such as packaging material for avoiding oxidation of sensitive food, thus expanding the spectrum of its uses.

The application of melanins for the modification of packaging polymers known from literature is relatively limited. Dong et al. modified poly(vinyl alcohol) with natural melanin from cuttlefish ink and synthetic melanin from dopamine hydrochloride autoxidation. Their results revealed that melanins may enhance the thermal stability of polymer, even in low content (0.5 mass%) due to their ability to scavenge radicals responsible for the degradation process [40]. Shanmuganathan et al. have reported that synthetic melanins derived from the oxidation of L-Dopa significantly improve the onset decomposition temperature of PMMA by 50–90 °C, when the addition of melanins amounts are low (0.5–5 mass%) [39]. Kiran et al. synthetized nanomelanin-polyhydroxybutyrate nanocomposite film which showed antioxidant activity and a strong protective effect against multidrug-resistant *Staphylococcus aureus* [41]. It is noteworthy that melanin itself may be used to prepare thin films [45,46,47].

In order to adequately preserve the quality of food goods, the packaging materials have to provide efficient barriers against light, water vapor, atmospheric gases and volatile organic compounds, preventing food spoilage. When the modified blend film is applied to preserve food, its integrity has to be maintained and external stress withstood, so these mechanical properties are vitally important characteristics of the film [1]. A relatively wide-ranging tensile strength (14–70 MPa) and deformation at break (1–8%) has been found, depending on the type of PLA and process [5]. Due to the lack of literature relating to the effect of melanin on the mechanical properties of PLA-based films, other non-thermoplastic substances were considered in order to discuss the results described above in this work. Rhim et al. reported that the addition of nanoclay (Cloisite 20A) had a negative impact on both, the tensile strength of the PLA-based films and elongation at break when the additive represented more than 3% (*w*/*w*) of the PLA/nanoclay composite film. Both parameters decreased explicitly with an increase of nanoclay content and eventually, when the PLA/Cloisite 20A ratio was 95:5, the values of tensile strength and elongation at break dropped by approximately 10% [11]. Jamishidan et al. described the influence of selected antioxidants on the mechanical and surface properties of PLA based bio-composites. Both α-tocopherol and ascorbyl palmitate reduced PLA tensile strength by about 8% and 43% respectively. α-tocopherol as a plasticizer, increased elongation at break by about 16% [48]. Our results suggest, that melanin in low and moderate concentrations may enhance the mechanical properties of PLA such as tensile and burst strength, whereas high melanin content may result in a deterioration of PLA properties. 

Dynamic mechanical analysis is a method based on oscillating loading of a sample and an elastic and viscous response monitored as a function of temperature. The results are expressed by three parameters: the storage modulus (*E*’), the loss modulus (*E*’) and the loss factor—tan δ (which is ratio *E*’’/*E*’). The storage modulus usually strongly decreases when temperature crosses the dynamic glass transition (when heating at constant frequency), when the loss modulus (*E*’’) and tan δ exhibit a peaked shape [10]. The DMA analysis for the films was carried out to determine the effect of the melanin on their thermomechanical properties. PLA is a semi-crystalline material, and its storage modulus decrease rapidly when the material enters its glass transition. Thus, it displays a region of relative stability before its modulus plummets rapidly as PLA structure approaches the melting point [10]. As can be seen in Figure 1B sample “0” and “2” shown a similar pattern, while the peak of sample “1” was more intense. On the contrary, the presence of the highest melanin content in sample “3” hinders the polymer chains mobility, resulting in a broader and larger peak in comparison to other samples. The different shape of the tan δ peak (Figure 1C) can be explained taking into account the random placement of the melanin particles within the PLA matrix. Indeed, this arrangement leads to the overlapping of the melanin particles, thus creating some matrix-poor areas, which can influence the PLA chain mobility [10,28].

Food products are very susceptible to rancidity caused by oxidation of lipids that contain unsaturated fatty acids that can be attacked by oxygen free radicals. Antioxidants are added to foods to intercept and react with these free radicals at a faster rate than the lipid substrate. Nevertheless, the current incorporation of antioxidants throughout the entire food matrix in one large initial dose is not an efficient process due to the oxidation occurring at the surface and high initial doses of antioxidant having a pro-oxidant effect. Therefore, one emerging technology is the use of antioxidant active packaging, where the antioxidant is incorporated to a packaging material with the purpose of being delivered to the food surface during commercialization, at an appropriate rate. Most of the active packaging developments base their work on the mass transportation properties of plastic materials (sorption, migration, and permeation), and the release of the active agents depends on several factors, such as the type of polymer and type of food [49]. However, the presence of synthetic antioxidants in food is questionable, owing to the potential risks. This has been encouraged by strong consumer demand, as synthetic compounds are frequently perceived as undesirable or harmful. Natural antioxidants are preferred to artificial substances, especially by consumers [36,37]. Moreover, the use of active antioxidant packaging that incorporates natural antioxidants presents important advantages. The addition of a natural compound to the packaging may reduce the need to use synthetic antioxidants in the plastic, reducing the risk of potential toxicity by migration [49]. Several reports of PLA modifications by antioxidants are known from the literature. A wide spectrum of additives was used including humic acids [42], α-tocopherol and ascorbyl palmitate [48,50], as well as nano-lignin [51]. Some other natural additives have also been used to modify other polymers to develop antioxidant activity, such as green tea extract [49], extracts from pine bark and grapes, carotenoid-containing oleoresin from tomato processing [52], extracts of red and white grape seeds and tomato [53].

Phenolic structures present in melanin molecules are considered to be major electron donating moieties and responsible for antioxidant activity [36,37]. A standard Folin-Ciocalteu method was utilized for this purpose, which is a simple technique extensively used in the quantification of phenolic compound content in various substances. Following the methodology described by Bishai et al. [42] the total available phenolics have been determined to be 0.018; 0.020 and 0.033 μmole GAE/g film for samples “1”, “2” and “3”, respectively. No polyphenolics were detected in pure PLA film (sample “0”), which is evident as PLA does not contain polyphenolics. It is comparable to the results of Bishai et al. who modified PLA with the incorporation of humic acid, and in their study, the total available phenolic was determined to be a 0.075 μmole GAE/g film [42]. Ambrogi et al. stated that the addition of polyphenolics containing compounds, such as extracts from pine bark and grapes and carotenoid-containing oleoresin from tomato processing increased the oxidative stability of polypropylene films [52]. Cerruti et al. also demonstrated that natural additives, such as extracts from red and white grape seeds and tomato effectively stabilize the polymer by acting as an antioxidant [53].

To evaluate the antioxidant properties of the films, methods based on the color quenching of synthetic radical ABTS and DPPH were performed in aqueous and methanol medium, respectively. Within this reaction charged ABTS chromophore is decolorized by electrons donated by hydroxyl/phenolic groups of melanin and a decrease in absorbance was measured at 734 nm. The DPPH method was based on the presence of its odd electron, DPPH offers strong absorption maxima at 517 nm (purple) with a visible spectroscopy. As the odd electron of the radical becomes paired off in the presence of a hydrogen donor, i.e., a free radical scavenging antioxidant, the absorption intensity is decreased. The resulting decolorization is stochiometric with respect to the number of electrons captured [36,37,42]. Modified PLA/melanin films have shown good radical scavenging activity. Our results are comparable to results obtained by Bishai et al. who used humic acid to develop antioxidant activity in PLA films [42].

Sensitive components of food such as lipids, flavors, vitamins and pigments may undergo photodegradation reactions. The spectrum and the intensity of the light source, the conditions of light exposure, and the degree of packaging material light transmittance are factors that can significantly affect food quality. Thus, packaging plays a pivotal role in the prevention of the photodegradation of food components during storage [1]. The design of the packaging for a specific food product involves not only the choice of appropriate packaging material, but also the addition of the right additives or stabilizers to the packaging in order to provide a more efficient UV-Vis light barrier, and thus a significant improvement in the protection of food quality after storage. The absorption and transmission of light by polymers is particularly important in the food packaging industry where the packaged goods are light sensitive. Another issue in fresh food packaging is the effect of irradiation in the package, since ultraviolet light irradiation is a common method used for lowering microbial population in foods [1]. PLA has reasonably good optical properties compared to existing petroleum-based polymers [1,54]. PLA reinforced with bentonite, layered silicate, and microcrystalline cellulose has shown promising results in terms of a reduction in UV transmission and visual radiation, which can be advantageous in packaging applications [1]. Shanmuganathan et al. have reported that the incorporation of melanins at very low levels (0.5–5 mass%) to PMMA, decreased the transmittance of material by 80% [39]. At 225 nm PLA shows a significant increase in UV light transmitted, reaching about 85% at 250 nm and 95% at 300 nm. Thus, most of the UV-B and UV-A radiation passes through the PLA films. No UV radiation transmission was found in the lower range of UV, in the 190–220 nm wavelength region. As shown in Figure 2 the addition of melanin into PLA produced approximate 8% improvement in UV-Vis barrier properties at 400 nm and approximately 7% at 280 nm. PLA the maximum absorbance wavelength is 240 nm and can be attributed to the ester group present in the polymer [1]. The melanin transmittance pattern is very similar to that of PLA, thus the UV-Vis barrier properties were not strongly enhanced, but nevertheless, some improvement was observed.

FT-IR and Raman spectroscopies are useful and highly important tools to characterize the physicochemical nature of the polymers [1,9,10,15]. Due to the high sensitivity of FT-IR spectroscopy to changes in the dipole moment of a given vibrating group, this technique was used to identify polar groups. In contrast to Raman spectroscopy, it is especially useful in the characterization of the homonuclear polymer backbone due to its sensitivity to changes and polarizability [1]. Peaks of 867.67 cm^−1^ and 754.23 cm^−1^ appear as two bands that can be attributed to the amorphous and crystalline phases of PLA, respectively, suggesting, that the films obtained may have both phases in their structure [1]. No evident differences between samples 0, 1 and 2 were observed. This may be caused by fact that FT-IR spectroscopy is a rather shallow penetrative technique (approximately 0.5 μm at 4000 cm^−1^ up to 5 μm at 400 cm^−1^) whereas film thickness was 75–80 μm. Consequently, the more penetrative Raman spectroscopy was used [55,56]. Results of the Raman spectroscopy analysis showed noticeable differences in the obtained spectra. With higher melanin content peaks were observed with greater insensitivity. The peaks can be interrelated as originating from the in-plane stretching of the aromatic rings and the linear stretching of the C–C bonds within the rings, along with some contributions from the C–H vibrations in the methyl and methylene groups in the melanin molecules [57]. A peak at 2000 cm^−1^ is similar to those obtained by Galvan et al. from eumelanin and may be caused by the stretching of three of the six C–C bonds within the melanin aromatic rings [58]. It was noted, that on all modified films, Raman spectra peaks at 395 cm^−1^ are present, which are thought to correspond to peaks obtained from pheomelanin and eumelanin and are caused by an out-of-plane deformation of the phenyl rings. Peaks at 2010 cm^−1^ are also similar to peaks seen in pheomelanin and are probably due to overtone or combination bands [57,58].

The water contact angle of the material is associated with its hydrophilicity. In general, the smaller the water contact angle, the higher the hydrophilicity. In the work of Cui et al. the water angle of pure PLA was approximately 72° [9]. In our work, the contact angle of pure PLA was approximately 66.96°. This discrepancy may be a result of the PLA compositions from various manufacturers. The addition of melanin into PLA at all concentrations did not significantly (*p* > 0.05) affected the surface properties of the polymer. In contrast, in a study by Jamishidan et al. the authors observed that ascorbyl palmitate decreased the PLA film contact angle (of a water droplet) and increased the polarity and wettability of the material [48]. 

One of the most important properties of bio-based films for the application of packaging is to minimize moisture transfer from the environment to the packed goods. Water vapor permeability (WVP) is one of the most important properties in food packaging due to the noticeable role water has in deteriorative reactions and microbial growth. For this purpose, the WVP of packaging materials should be as low as possible [1,16,20,59]. Jamishidan et al. observed that pure PLA films had parameters that were minimally lower than compared to the films containing either 2% of α-tocopherol or 1% of ascorbyl palmitate. However, no significant differences between the values were reported [48]. Rhim et al. proved that the incorporation of Cloisite 20A nanoclay decreased the values by less than 3% [11]. In our study, the addition of 0.025% of melanin into the PLA matrix resulted in improved barrier properties. This may be caused by the intramolecular interactions of the melanin particles and PLA chains. However, at higher concentrations (0.05% and 0.2%), the WVTR of modified blends was exacerbated than compared to neat PLA. This might be attributed to the fact that the addition of some compounds in high concentrations may increase the average pore size of the films, thus facilitating water molecule penetration into the polymer matrix [16].

Some additives for PLA composites may influence the color values of the modified blends. Li et al. developed antimicrobial packaging film made from PLA with TiO_2_ and Ag nanoparticles. The addition of TiO_2_ increased the L* (lightness) value of the modified films, due to the white color of nano-TiO_2_ powder, but in contrast, the addition of Ag nanoparticles resulted in a dark color explained by the argentous sheen of the nano-Ag powder [16]. Liu et al. noted that the addition of oregano essential oil had a clear influence on the color values of the PLA films [20]. Shi et al. observed that the co-polymerization of PLA with 3,4-dihydroxyphenylalanine, an amino acid being a precursor of DOPA melanin resulted in a yellow-brown color in the modified films [60]. Also Kiran et al. observed that synthetized nanomelanin-polyhydroxybutyrate nanocomposite film is characterized by a yellowish-brownish color [41]. Our results indicate that the increasing addition of melanin influences the color values in comparison to pure PLA film, leading to reduction in lightness (L*), as well as an increase in the redness (a*) and yellowness (b*) values. ∆*E* values ranged from 0.60 to 1.33. ∆*E* > 1 is considered perceptible to the human eye, so the highest melanin content caused noticeable color changes. The yellowness (*YI*) increased with increasing melanin amount, while the whitening index (*WI*) decreased when the melanin content was increased. The yellowness index or a change in the degree of yellowness is a number calculated from spectrophotometric data that describes the change in color of a test sample from clear or white to yellow. The opacity of PLA/melanin films decreased with the addition of melanin particles (from 6.90 ± 0.09 of sample “0” to 6.41 ± 0.12 of sample “3”). This was probably due to the color of the melanin powder. The changes of film transparency as a consequence of the addition of nanoparticles had been reported with PLA films [15]. However, the difference in opacity among film samples was not perceptible to the human eye and not statistically significant (*p* > 0.05). PLA/melanin films in all melanin concentrations still had good transparency, even at high melanin content. This result suggested high PLA/melanin film transparency, meaning that packaging film could be transparent, which an important requirement for consumers and this would have a clear influence on customer choice [16].

Compared to the neat PLA film, the addition of melanin particles into polymer matrix did not affect the *T*_g_ and *T*_m_ of PLA. This might be because the addition of the melanin did not change the mobility of the PLA macromolecular chains [16,20]. On the other hand, it can be seen in Figure 7, that the cold crystallization temperature (*T*_c_) was changed by the introduction of melanin and led to an obvious decrease in comparison to pure PLA. Similar observations were made by Li et al. [16] who found that the addition of titanium dioxide and silver nanoparticles did not result in obvious changes in thermal transitions of PLA, but introduction of nano-TiO_2_ decreased the *T*_c_ values of modified films. As can be seen in Figure 7, the addition of melanin changed the melting behavior of resulted films in comparison to pure PLA. Under this heating conditions, the amorphous PLA chains do not have enough time to self-adjust, resulting in reduced cold crystallization and the subsequent multi-melting behavior, which is indicative of polymorphism, melt recrystallization [61] or to lamellar populations with different perfection degrees [62]. As can be seen in Figure 7 the enthalpy of crystallite formation in cold crystallization temperature is in fact almost identical to the melting enthalphy of crystallites at the melting point, which means that all crystallites in the polymer matrix rise above approximately 100 °C, which is confirmed by the cooling curve, where there is no peak of crystallization, only the glass transition temperature. As listed in Table 4, the addition of melanin led to a significant increase of the χ values of modified PLA/melanin films in comparison to the PLA film devoid of melanin. Crystallization degrees of pure PLA and PLA/melanin films values are relatively low indicating, that all the tested samples presented high amorphic structure. According to Li et al. this result can be explained by the phenomenon of heterogeneous nucleation [16]. Considering the increase in χ and decrease in the cold crystallization peaks as a result of melanin addition it is tempting to suggest that melanin is acting as a nucleating agent. Sullivan et al. observed that cellulose nanocrystals also can act as nucleating agent when added to PLA [61], whereas Bishai et al. [42] noticed that incorporation of humic acid into PLA matrix reduced its crystallinity.

Modified PLA/melanin films showed antibacterial activity against *E. faecalis*, *P. aeruginosa* and *P. putida*. No antibacterial activity towards, *E. coli* and *S. aureus* was observed. This data are supported by previous study showed that melanins from some fungi have been active against *P. aeruginosa* and *E. faecalis* [36,37]. The literature on melanin applications to develop antimicrobial properties of polymer film is relatively limited. Kiran et al. synthetized nanomelanin-polyhydroxybutyrate nanocomposite film which showed a strong protective effect against multidrug-resistant *Staphylococcus aureus* [41]. Kuang et al. developed antimicrobial DOPA-melanin coatings that were able to load and release of a cationic aminoglycoside active against *S. aureus* [45]. Some authors noted antimicrobial activity of melanins from various microbial sources. Helan Soundra Rani et al. [63] noted the antimicrobial activity of melanin isolated from halophilic black yeast *Hortaea werneckii*. Laxmi et al. [64] observed that growth of *P. aeruginosa* was inhibited on the presence of melanin obtained from *Providencia rettgeri*. Xu et al. [65] analyzed the antimicrobial activity of melanin from *Lachnum* YM30 and noted that it was active against a wide spectrum of bacteria, including *S. aureus*. The authors suggest that melanin antibacterial activity might result from damage of the cell membrane and affect bacteria membrane function. A discrepancy in melanin antimicrobial activity may result in differences within the molecule structure and composition but also particles size [41,66]. On the other hand, there are some reports that melanins have antibiofilm activity against pathogenic bacteria including *P. aeruginosa* and could interfere with bacterial quorum-sensing system, regulate its associated functions, and prevent bacterial pathogenesis [64,65,67,68].

## 5. Conclusions

This article explored the properties of modified PLA films incorporated with fungal melanin. The properties of PLA/melanin composites were compared to pure PLA. Melanin played a vital role in mechanical, antioxidant, antimicrobial and barrier properties. The UV-Vis barrier properties of modified PLA were slightly improved by the incorporation of melanin. However, tensile and burst strength, water vapor transmission rate, color and the antioxidant properties of PLA based films varied depending on the presence and amount of melanin. An improvement in the antioxidant activities of modified PLA film is worth mentioning as an important aspect of the work. Considerable improvement in these properties has been observed in comparison to pure PLA. The results described here are particularly interesting if one considers that the additives used have a natural origin and are extracted from bio-waste, providing added value in the development of sustainable alternatives to traditional synthetic antioxidants.

## Figures and Tables

**Table 1:** Tensile strength in machine direction (TS MD), tensile strength in transversal direction (TS TD), burst strength (BS), seal strength (SS) and Water Vapor Transmission Rate (WVTR) of pure PLA and PLA/melanin modified films.

Sample	TS MD (MPa)	TS TD (MPa)	BS (MPa)	SS (MPa)	WVTR (g/(m^2^ × Day))
0	59.47 ± 8.86	45.87 ± 1.01	23.50 ± 2.41	10.18 ± 1.68	24.60 ± 0.33
1	63.40 ± 4.30	61.55 ± 3.23	27.45 ± 1.45	8.75 ± 2.35	21.30 ± 2.56
2	63.78 ± 5.16	54.87 ± 2.56	23.78 ± 1.59	8.92 ± 0.92	23.60 ± 0.98
3	45.42 ± 2.84	40.78 ± 1.04	16.10 ± 2.92	7.12 ± 0.77	28.20 ± 1.31

**Table 2:** Color parameters (L*, a*, b*), Δ*E*, yellowness index (*YI*), whitening index (*WI*) and opacity of pure PLA and PLA/melanin modified films.

Sample	L*	a*	b*	Δ*E*	*YI*	*WI*	Opacity
0	97.41 ± 0.00	0.01 ± 0.00	0.21 ± 0.01	used as standard	0.31	97.40	6.90 ± 0.09
1	97.03 ± 0.00	0.05 ± 0.00	0.67 ± 0.00	0.60	0.99	96.95	6.67 ± 0.04
2	97.02 ± 0.00	0.07 ± 0.00	0.78 ± 0.00	0.69	1.15	96.92	6.53 ± 0.07
3	96.51 ± 0.01	0.18 ± 0.00	1.18 ± 0.00	1.33	1.75	96.31	6.41 ± 0.12

**Table 3:** The antioxidant activity determined by ABTS (*AA*% ABTS) and DPPH (*AA*% DPPH) methods and available phenolic groups (APG) of pure PLA and PLA/melanin modified films.

Sample	*AA*% ABTS (%)	*AA*% DPPH (%)	APG (μmole GAE/g)
0	0.00 ± 0.00	0.00 ± 0.00	0.00 ± 0.00
1	5.83 ± 0.11	5.69 ± 0.04	0.0181 ± 0.006
2	11.41 ± 0.23	7.43 ± 0.12	0.0205 ± 0.013
3	23.20 ± 0.09	21.66 ± 0.15	0.0234 ± 0.009

**Table 4:** Thermal characteristics of pure PLA and PLA/melanin films.

Sample	*T*_g_ (°C)	*T*_c_ (°C)	*T*_m_ (°C)	*χ* (%)
0	61.4	112.9	148.7	0.23
1	61.2	107.1	147.4	0.92
2	61.5	108.3	147.9	2.43
3	61.5	107.3	147.5	1.61
